# Genomic and cytogenetic analysis of the *Ceratitis capitata temperature-sensitive lethal* region

**DOI:** 10.1093/g3journal/jkad074

**Published:** 2023-03-29

**Authors:** Germano Sollazzo, Georgia Gouvi, Katerina Nikolouli, Roswitha A Aumann, Haig Djambazian, Mark A Whitehead, Pierre Berube, Shu-Huang Chen, George Tsiamis, Alistair C Darby, Jiannis Ragoussis, Marc F Schetelig, Kostas Bourtzis

**Affiliations:** Insect Pest Control Laboratory, Joint FAO/IAEA Centre of Nuclear Techniques in Food and Agriculture, Friedensstrasse 1, 2444 Seibersdorf, Austria; Department of Insect Biotechnology in Plant Protection, Justus-Liebig-University Gießen, Institute for Insect Biotechnology, Winchesterstr. 2, 35394 Gießen, Germany; Insect Pest Control Laboratory, Joint FAO/IAEA Centre of Nuclear Techniques in Food and Agriculture, Friedensstrasse 1, 2444 Seibersdorf, Austria; Laboratory of Systems Microbiology and Applied Genomics, Department of Sustainable Agriculture, University of Patras, 2 G. Seferi St., 30100, Agrinio, Greece; Insect Pest Control Laboratory, Joint FAO/IAEA Centre of Nuclear Techniques in Food and Agriculture, Friedensstrasse 1, 2444 Seibersdorf, Austria; Department of Insect Biotechnology in Plant Protection, Justus-Liebig-University Gießen, Institute for Insect Biotechnology, Winchesterstr. 2, 35394 Gießen, Germany; McGill University Genome Centre, McGill University, Montreal, QC H3A 0G4, Canada; Centre for Genomic Research, Institute of Integrative Biology, The Biosciences Building, Crown Street, L69 7ZB Liverpool, UK; McGill University Genome Centre, McGill University, Montreal, QC H3A 0G4, Canada; McGill University Genome Centre, McGill University, Montreal, QC H3A 0G4, Canada; Laboratory of Systems Microbiology and Applied Genomics, Department of Sustainable Agriculture, University of Patras, 2 G. Seferi St., 30100, Agrinio, Greece; Centre for Genomic Research, Institute of Integrative Biology, The Biosciences Building, Crown Street, L69 7ZB Liverpool, UK; McGill University Genome Centre, McGill University, Montreal, QC H3A 0G4, Canada; Department of Insect Biotechnology in Plant Protection, Justus-Liebig-University Gießen, Institute for Insect Biotechnology, Winchesterstr. 2, 35394 Gießen, Germany; Insect Pest Control Laboratory, Joint FAO/IAEA Centre of Nuclear Techniques in Food and Agriculture, Friedensstrasse 1, 2444 Seibersdorf, Austria

**Keywords:** Mediterranean fruit fly, sterile insect technique, genetic sexing strain, white pupae, Tephritidae

## Abstract

Genetic sexing strains (GSS) are an important tool in support of sterile insect technique (SIT) applications against insect pests and disease vectors. The yet unknown *temperature-sensitive lethal* (*tsl*) gene and the recently identified *white pupae* (*wp*) gene have been used as selectable markers in the most successful GSS developed so far, the *Ceratitis capitata* (medfly) VIENNA 8 GSS. The molecular identification of the *tsl* gene may open the way for its use as a marker for the development of GSS in other insect pests and disease vectors of SIT importance. Prior studies have already shown that the *tsl* gene is located on the right arm of chromosome 5, between the *wp* and *Zw* loci (*tsl* genomic region). In the present study, we used genomic, transcriptomic, bioinformatic, and cytogenetic approaches to characterize and analyze this genomic region in wild-type and *tsl* mutant medfly strains. Our results suggested the presence of 561 genes, with 322 of them carrying SNPs and/or insertion–deletion (indel) mutations in the *tsl* genomic region. Furthermore, comparative transcriptomic analysis indicated the presence of 32 differentially expressed genes, and bioinformatic analysis revealed the presence of 33 orthologs with a described heat-sensitive phenotype of *Drosophila melanogaster* in this region. These data can be used in functional genetic studies to identify the *tsl* gene(s) and the causal mutation(s) responsible for the temperature-sensitive lethal phenotype in medfly, and potentially additional genes causing a similar phenotype.

## Introduction

The Mediterranean fruit fly (medfly) *Ceratitis capitata* (Wiedemann) is a major agricultural insect pest in almost all continents (Harris and Lee 1989). The sterile insect technique (SIT) is an environment-friendly, species-specific method, which is used as a component of area-wide integrated pest management strategies for the population control of medfly as well as other insect pests and disease vectors (Knipling 1955; Enkerlin 2003; Dyck *et al*. 2021; Klassen and Vreysen 2021). SIT is based on the mass production, sterilization by irradiation, and systematic release of sterile insects over an area to suppress, locally eradicate, contain, or prevent the (re)establishment of the SIT-targeted insect pest populations (Enkerlin *et al*. 2017; Dyck *et al*. 2021; Klassen and Vreysen 2021). Although many, and successful, SIT programs have been based on bisexual releases, male-only releases are more efficient and cost-effective, as has been shown in medfly, and can be achieved using genetic sexing strains (GSS) (Hendrichs *et al*. 1995; Rendon *et al*. 2004; Franz *et al*. 2021).

VIENNA 7 and VIENNA 8 are the two most commonly used GSS in SIT applications against medfly (Augustinos *et al*. 2017). They are based on two selectable markers, the *white pupae* (*wp*) and the *temperature-sensitive lethal* (*tsl*) genes (Franz *et al*. 2021). Irradiation-induced translocations have linked the wild-type alleles of these genetic loci with the male-determining region (*MoY*) on the Y-chromosome (Meccariello *et al*. 2019; Franz *et al*. 2021), and cytogenetic analysis identified the translocation breakpoints of VIENNA 7 and VIENNA 8 GSS on trichogen polytene chromosomes at T(Y; 5)52A and T(Y; 5)58B, respectively (Zacharopoulou *et al*. 2017; Franz *et al*. 2021). Therefore, males of these GSS are heterozygous for these loci, emerge from brown puparia and are resistant to elevated temperatures. At the same time, females are homozygous for the mutant alleles of the *wp* and *tsl* genes, emerge from white puparia and are sensitive (die) when exposed at high temperatures, 34°C or 35°C (Caceres 2002; Augustinos *et al*. 2017; Franz *et al*. 2021).

More than 20 years of research and development efforts were required to construct and validate VIENNA 8 GSS, the most successful and high-quality GSS developed so far. Although there has been an increase in requests by FAO and IAEA Member States to develop and implement the SIT against different insect plant pests, livestock pests, and disease vectors, for most of them, there is no sexing system which could be used in support of such applications. Therefore, a “*generic approach*” is needed for the prompt development of GSS, which will not depend on the random discovery or induction of mutations which could be used as selectable markers for sex separation. The suggested concept is based on the discovery of genes which are useful markers for genetic sexing strategies, the characterization of the causal mutations, the identification of the orthologous genes in SIT-targeted species, and finally the induction of the same or similar mutations using genome editing approaches (FAO/IAEA 2021).

The availability of genomes, transcriptomes, genome editing, and functional genetics tools for many of the SIT-targeted insect pest species and disease vectors can greatly support the concept of the “*generic approach*” (Sim *et al*. 2019; FAO/IAEA 2021). However, the (cyto-)genetic characterization of the successfully used medfly GSS, and especially the molecular identification of its marker genes (*wp, tsl*) are key factors to facilitate the concept. Genetic studies and in situ hybridization experiments allowed the mapping of several genes and the establishment of linkage groups (Brown *et al*. 1989; Konsolaki *et al*. 1990; Tolias *et al*. 1990; Rina and Savakis 1991; Zacharopoulou *et al*. 1992; Scott *et al*. 1993; Zwiebel *et al*. 1995; Gariou-Papalexiou *et al*. 2002; Stratikopoulos *et al*. 2008). Mapping the genes *white*, *yellow*, *PS_2_a*, *Pgd*, *S36*, *S38*, *Vg1*, *Vg2*, *Sxl*, and *Zw* on chromosome 5 in medfly indicated synteny to the *Drosophila melanogaster* X chromosome (Gariou-Papalexiou *et al*. 2002; Zacharopoulou *et al*. 2017). Earlier studies, which were based on deletion and transposition mapping combined with cytogenetic analysis, suggested that the medfly *wp* and *tsl* genes are located at position 59B and 59B–61C of the trichogen polytene chromosome map, respectively (Kerremans and Franz 1994). The chromosomal inversion D53 was also found in the vicinity of these genes as it spans the region 50B–59C of the medfly chromosome 5 [on trichogen polytene chromosome map; (Zacharopoulou *et al*. 2017; Franz *et al*. 2021)]. Inversion D53, which has been used as a recombination suppressor in VIENNA 8 GSS to enhance its genetic stability, played a major role in identifying the gene responsible for the white puparium phenotype. Comparative genomic analysis of strains with and without the inversion led to the identification of the right breakpoint of this chromosomal alteration. Combined with comparative transcriptomic analysis, it resulted in the discovery of the *wp* gene located inside the inversion, close to its right breakpoint (Ward *et al*. 2021). In situ hybridization analysis localized the *wp* gene on position 76B of the salivary gland polytene chromosomes, which corresponds to position 59B of trichogen polytene chromosomes, thus confirming the results of the deletion mapping experiments (Ward *et al*. 2021). The discovery of the *wp* gene and the mutations responsible for the white puparium phenotype in medfly and two closely related tephritid species (the Oriental fruit fly *Bactrocera dorsalis* and the melon fly *Zeugodacus cucurbitae*) allowed the induction of CRISPR/Cas9 mutations in the orthologous *wp* gene of the Queensland fruit fly *Bactrocera tryoni* and the establishment of the respective mutant line, thus providing proof-of-concept for the “*generic approach*” (Ward *et al*. 2021).

Although the *wp* gene is a useful selectable marker, it only allows sex separation at the pupal stage. In contrast, the *tsl* gene can be used to separate males and females earlier in development, at the embryonic stage. Therefore, the discovery of this gene would provide a powerful tool for the generic approach to develop GSS for SIT applications. Unlike the white pupae phenotype for which we had functional proof that it is due to a single-copy gene (Ward *et al*. 2021), such evidence is not available for the tsl phenotype; it is unknown whether it is due to a single (protein coding or not) gene or multiple genes, closely linked or not. The *wp* mutation was isolated during an irradiation experiment (Rössler 1979) and it was recently discovered that the white pupae phenotype is due to the insertion of a transposable element (Ward *et al*. 2021). On the other hand, the *tsl* mutation was isolated during an ethyl methanesulphonate (EMS) screen (Busch-Petersen 1990). Both irradiation and EMS mutagenesis screens may induce numerous and various types of mutations, from single nucleotide substitutions to large chromosomal rearrangements, making the detection of a mutation associated with a certain phenotypic trait a rather challenging task. Based on all the currently available genetic and cytogenetic information, the *tsl* gene(s) is located downstream of *wp*, in the region 59B–61C of the trichogen polytene chromosome map, which most likely corresponds to the area 76B–78C of the salivary gland polytene chromosome map (Kerremans and Franz 1994; Niyazi *et al*. 2005; Franz *et al*. 2021). As the *glucose-6-phosphate 1-dehydrogenase* gene (also known as *Zw*) has been located, by in situ hybridization, on position 79C of the polytene chromosome map (Scott *et al*. 1993), the *tsl* gene is certainly located inside the genomic region between *wp* and *Zw* (hereafter *tsl* genomic region), which corresponds to 76B–79C on the map of the medfly polytene chromosome 5. In situ hybridization has been used in this study to localize candidate *tsl* genes as well as to validate the assembly of the *tsl* genomic region. The present study uses cytogenetic, genomic, transcriptomic, and bioinformatic approaches to characterize the medfly *tsl* genomic region and to identify polymorphisms that could potentially be associated with the tsl phenotype toward the discovery of the *temperature-sensitive lethal* (*tsl*) gene.

## Materials and methods

### *Ceratitis capitata* strains and rearing conditions

Two wild-type strains, Egypt II (EgII) and Benakeion, the VIENNA 7 GSS, and the *wp tsl* mutant strain of medfly were used in the present study. All four strains were kept under standard laboratory conditions at 24 ± 2°C, 55 ± 10% RH, and 14/10 h light/dark cycle. Adults were fed on yeast and sugar (1:3) with water being provided separately, while larvae were reared on a carrot diet as described previously (Sollazzo *et al*. 2022).

### Transcriptomic analysis

*Ceratitis capitata* Benakeion and *wp tsl* strains were used in a preliminary experiment to estimate the time required for eggs to be exposed at 34°C for the expression of the tsl phenotype (lethality). Egg collections were performed from cages at their fifth to eighth oviposition day. Eggs were counted and placed on black filter papers (three replicates of 100 eggs per filter paper) on top of ca. 50 g of carrot diet, in 90 × 15 mm Petri dishes. The latter were kept at 25°C for 24 h and then incubated for 30, 60, 120, 240, 360 min and 24 h at 34°C. Control plates remained at 25°C. Egg hatching was estimated five days after the egg collection. Based on the results, two time points (60 and 120 min) were chosen for the transcriptomic analysis and 150 eggs were placed per filter paper. Three replicates per strain, temperature and time point were performed. Eggs were kept at 25°C for 24 h and then incubated for 60 and 120 min at 34°C, while the control plates were left at 25°C for 60 min. RNA extraction was performed after the end of each exposure period. Total RNA was extracted by homogenizing each pool of 150 eggs in liquid nitrogen and using the RNeasy Micro kit (QIAGEN).

mRNA was isolated using the NEBNext polyA selection and the Ultra II directional RNA library preparation protocols from NEB and sequenced on the Illumina Novoseq 6000 at the Centre for Genomic Research, Institute of Integrative Biology, University of Liverpool, UK, using dual indexes as 150 bp paired-end reads (library insert 500 bp). Individual libraries were sequenced to provide >1 million paired-end reads per sample. Each replicate was then assembled separately using Trinity (Haas *et al*. 2013). The assembled transcripts from Trinity were mapped to the Ccap 3.2.1 genome (accession GCA_905071925.1) using minimap (Li 2018) (parameters -ax splice:hq -uf). The Illumina reads were mapped with STAR (Dobin *et al*. 2013). The freely available open-source software Integrative Genomics Viewer (IGV) v 2.6 (Robinson *et al*. 2011) was used to view all data at a genomic and gene level as well as to manually search variants/polymorphisms inspecting gene-by-gene the whole region. The annotation of the Ccap 3.2.1 genome was performed with Funannotate (v1.6.0) (Palmer and Stajich 2020). The annotation was generated during a project to identify *white pupae* genes of several tephritids (Ward *et al*. 2021) (see ENA bioproject PRJEB36344). Genomic repeats were identified using RepeatModeler (v.1.0.11) (Smit *et al*. 2008), and softmasked using RepeatMasker (v4.0.7) (Smit *et al*. 2013). The Funannotate pipeline comprises of multiple steps; (1) “train”—where a genome-guided transcriptome is generated using Trinity (v2.8.5) (Grabherr *et al*. 2011), (2) “predict”—which runs ab initio and evidence-based gene prediction tools, followed by consensus-based gene model selection, and (3) “update”—which is used to refine UTRs and gene models.

Differential expressed (DE) genes analysis between the wild-type and *wp tsl* mutant strains was assessed using the *edgeR* (Robinson *et al*. 2010) package in the Degust public server (https://degust.erc.monash.edu-v4.2-dev, Powell) with the False Discovery Rate cutoff set at 0.05 and abs logFC at 1.

### Genomic analysis and detection of polymorphisms

Genomic DNA from one male and one female of the *wp tsl* mutant strain were extracted using ExtractMe DNA tissue kit (Blirt, Poland) following the manufacturer's recommendations, while genomic DNA for the VIENNA 7 female was extracted using phenol/chloroform (Saccheri and Bruford 1993). The libraries for the *wp tsl* strain were prepared using an Illumina TruSeq Nano DNA Kit (Illumina, USA). They were sequenced on an Illumina HiSeq X platform resulting in 150-bp paired-end reads (Macrogen, Korea). The libraries for the VIENNA 7 GSS were prepared using an Illumina TruSeq PCR-free kit (Illumina, USA) and were sequenced on an Illumina HiSeq 400 platform resulting in 150-bp paired-end reads (Centre for Genomic Research, Liverpool). Raw fastq NGS reads were imported in Geneious Prime 2022.1.1 and subjected to trimming using BBDuk Adapter/Quality Trimming Version 38.37 with default settings (plug-in available on Geneious Prime 2022.1.1). The *wp tsl* trimmed reads were mapped to the reference sequence (*wp–Zw* region) extracted from Ccap 3.2.1 genome (accession GCA_905071925.1) using the “*Bowtie2” (v. 2.4.5*—*© Ben Langmead)* Geneious Prime 2022.1.1 plug-in with default parameters (*Alignment type: end to end; High sensitivity*). The obtained consensus contig for the *wp tsl* strain was used for polymorphisms calling.

A putative *tsl* polymorphism must be homozygous for the *tsl* allele in the *tsl* mutant strains (*wp tsl* males and females, and VIENNA 7 females). For this purpose, the Geneious Prime 2022.1.1 tool “*Find Variations/SNPs*” was used and polymorphisms meeting the following criteria were considered: (1) located inside and outside the coding sequence (Geneious Prime 2022.1.1 function), (2) had a minimum variant frequency of 0.80%, (3) had a minimum variant *P*-value of 10^−6^ (where *P*-value represents the probability of a sequencing error resulting in observing bases with at least the given sum of qualities), and (4) had a minimum strand bias *P*-value of 10^−5^ when exceeding 65% strand bias. Polymorphic regions found were classified as insertion, deletion, and SNPs. SNPs found in coding regions were classified as transition or transversion and as synonymous or nonsynonymous according to the nature of the nucleotide and amino acid change, respectively (Geneious Prime function).

For 10X genome sequencing, high molecular weight DNA was prepared as follows: 20 individuals of each sex and strain (EgII and *wp tsl)* were pooled, ground in liquid nitrogen and DNA was extracted using the QIAGEN Genomic tip 100/G kit (Qiagen, Germany). To remove smaller DNA molecule, we performed a size selection with 0.75% agarose SAGE BluePippin cassette (SAGE, MA, USA). The DNA samples were then processed into Linked-Read libraries using the single molecule barcoding kit from 10× Genomics Technologies. The method is based on gel beads containing DNA primers that contain part of the Illumina sequencing adapter (P5 side), bead barcode sequence, and a random sequence. DNA was joined with beads in an oil-buffer emulsion using a microfluidic system. The system was calibrated to produce a ratio close to 20:1 DNA per bead. The emulsion is then isothermally amplified using the bead primers. This process links the barcode to the synthesized strands. We then break the emulsion and perform cleanup, SPRIselect, end-repair, A-tailing, and ligation of the second part of the adapter containing library index and Illumina P7 sequence. After library QC and normalization, sequencing was performed on Illumina HiSeq X in paired-end with 150 cycles pooling two libraries per lane, yielding on average 200 million read pairs per library. The demultiplexed fastq files were assembled into contigs using the supernova assembler (version v2.1.1, 10X Genomics). Different molecules or GEM barcode fractions were tested, ranging from 0.4 to 1, with raw coverages varying between 56X and 120X. The representative assembly for each sample was chosen based on the highest N50. Optimal parameters were determined to be between 0.8 and 1 for barcode fraction, with raw coverage between 100X and 120X. Transcriptomic and genomic data used in the present study have been deposited to NCBI under BioProject No. PRJEB57574.

SNPs found in DE genes, and the medfly orthologs of the *D. melanogaster* heat-sensitive genes (see below) were confirmed through MEGABLAST searches against the EgII and *wp tsl* mutant strain 10X Genomics data using Geneious Prime 2022.1.1 BLAST tool. 10X Genomics data were previously set as a database and results were shown as “*Hit table including Query-centric alignment”*. Furthermore, a maximum of 100 hits per read was allowed with an E-value of 1e-100 and a scoring (match–mismatch) of 1–2.

### *Drosophila melanogaster* heat-sensitive genes and *Ceratitis capitata* orthologs

As the medfly temperature-sensitive lethal (tsl) phenotype is essentially a heat-sensitive phenotype since it is being expressed at elevated temperatures, we compiled a list of *Drosophila melanogaster* (*Dm*) temperature-sensitive genes by searching FlyBase (www.flybase.org) using the key word “*heat sensitive*”. Protein sequences were downloaded using FlyBase “*Sequence Downloader”* tool, imported in Geneious Prime 2022.1.1 and organized based on chromosomal location. Their medfly ortholog genes in the *tsl* genomic region were identified *via* blastp analysis (using default settings) and, using the reference sequence of this region (*wp–Zw* region), extracted from *C. capitata* 3.2.1 genome (accession GCA_905071925.1) as a database. The detected *Dm* heat-sensitive gene orthologs in the medfly *wp–Zw* region were screened for polymorphisms using the *wp tsl* consensus contig.

### In situ hybridization analysis

As reported previously, salivary glands from third-instar larvae were used as a source for polytene chromosome preparations for in situ hybridization analysis (Zacharopoulou 1990; Gouvi *et al*. 2022). The dissection of the glands was performed in 45% acetic acid, and the glands were then transferred on a cover slip in a drop of 3:2:1 fixative solution of glacial acetic acid/water/lactic acid. Once the glands became transparent, they were squashed. The quality of the polytene chromosomes was then checked in a phase contrast microscope and, if satisfactory, they were flattened via an overnight incubation at −20°C. Next day, the slides were frozen in liquid nitrogen, and the coverslip was removed with a razor blade. The final steps included dehydration of the polytene chromosome preparations in absolute alcohol, air-drying, and storage at room temperature (RT) until their use. DNA probes were prepared using Platinum Green Hot Start PCR Master Mix (2X; Thermo Fisher), while amplicon purifications were made with Zymo DNA Clean and Concentrator kit (Zymo Research, Orange, California). The primers used for the PCRs probes synthesis are presented in Supplementary Table 1. The probes were labeled with digoxigenin using the Dig DNA labeling Kit by Roche, according to the manufacturer's instructions. The DNA probes (120 ng in 15 µl final volume) were denatured for 10 min at 95°C, immediately put on ice and then mixed with 2 µl Hexanucleotide Mix 10×, 2 µl dNTP Labelling Mix and 1 µl Klenow enzyme labeling grade. The reactions were placed overnight at 37°C and were ended with the addition of 2 µl of 0.2 M EDTA (pH 8.0), 5 µl of distilled water, 25 µl 20× SSC, and 50 µl of HD formamide. The probes were stored at −20°C until their use.

In situ hybridization was performed as described in Ward *et al*. (2021). Before hybridization, stored chromosome preparations were hydrated subsequently for 2 min each in the following solutions: 70, 50, 30 ethanol, and then placed in 2× SSC at RT for 2 min. The stabilization of the chromosomes was done in 2× SSC at 65°C for 30 min, followed by denaturation in 0.07 M NaOH for 2 min, and washed in 2× SSC for 30 s. The slides were then dehydrated for 2 min in 30, 50,70, and 95% ethanol solutions, respectively, and air dried. The hybridization was performed on the same day by adding 15 µl of denatured probe (boiled for 10 min and ice-chilled). The slides were covered with siliconized coverslips, sealed, and incubated at 45°C overnight in a box laid out with wet Whatman paper to keep the preparations moist. After incubation, the coverslips were floated off in 2×SSC, and the slides were washed in 2×SSC for 3 × 20 min at 53°C, followed by one wash for 5 min in Buffer 1 (100 mM Tris–HCl pH 7.5/1500 mM NaCl). Slides were then placed for 30 min into Blocking Solution (Blocking reagent 0.5% in Buffer 1) followed by an additional wash for 1 min in Buffer 1. The antibody mix was then added to each slide and incubated in a box with wet Whatman paper for 45 min at RT. Two 15 min washes in Buffer 1 were followed by one wash for 2 min in Detection Buffer (100 mM Tris–HCl pH 9.5/100 mM NaCl). The color reaction was then started by adding 1 ml of NBT/BCIP solution to each slide and incubating for 40 min in the dark at RT. The removal of the NBT/BCIP solution was done with two washes for 1 min each in distilled water. Hybridization sites were identified and photographed using 60× and 100× oil objectives (bright field), with reference to medfly salivary gland chromosome maps (Zacharopoulou 1990).

## Results

### The *Ceratitis capitata* putative *tsl* region

The *tsl* gene is located in the *wp–Zw* (*tsl*) genomic region, on the right arm of chromosome 5 (Kerremans and Franz 1994; Niyazi *et al*. 2005). According to the recently sequenced genome of the medfly EgII strain (*Ccap3.2.1*—accession GCA_905071925.1), both *wp* and *Zw* genes are located on scaffold 5, which represents chromosome 5 (Ward *et al*. 2021). The *wp* gene is at position 61,551,139, and the *Zw* gene (*glucose-6-phosphate 1-dehydrogenase I*) is at position 67,751,599. The *tsl* genomic region is 6,200,460 bp long and contains 561 putative genes (Funannotate genome annotation), averaging 45 genes/500 kb and 160 exons/500 kb.

### RNA-Seq and DE genes

**Fig. 1.**
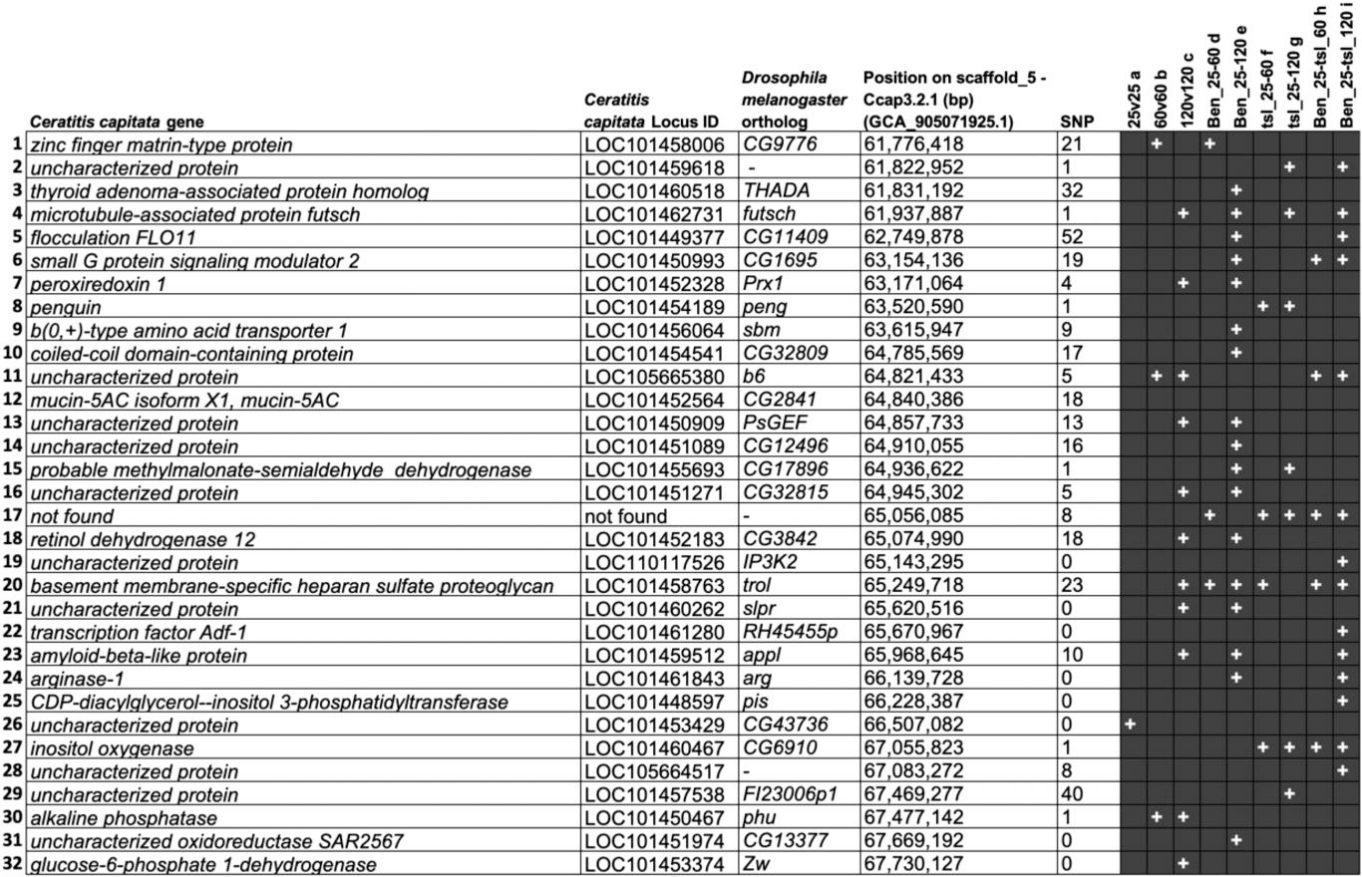
*Ceratitis capitata* differentially expressed genes located in the *tsl* genomic region. The *Ceratitis capitata* (*Cc*) gene name and locus, the *Drosophila melanogaster* (*Dmel*) ortholog name, the position in the reference genome sequence of medfly chromosome 5, the number of SNPs found in the coding region of the respective gene in the *wp tsl* mutant strain and the DE results for each gene are shown. All SNPs detected in the transcriptomic data were confirmed by the 10X genome sequence data of the *wp tsl* mutant strain.^+^ = differentially expressed; medfly strain names: “Benakeion” and “*wp tsl* mutant”; a, comparison of expression levels of Benakeion and *wp tsl* mutant kept at 25°C; b, comparison of expression levels of Benakeion and *wp tsl* mutant kept at 34°C for 60 min; c, comparison of expression levels of Benakeion and *wp tsl* mutant kept at 34°C for 120 min; d, comparison of expression levels of the Benakeion strain kept at 25°C and heat-shocked at 34°C for 60 min; e, comparison of expression levels of the Benakeion strain kept at 25°C and heat-shocked at 34°C for 120 min; f, comparison of expression levels of the *wp tsl* mutant strain kept at 25°C and heat-shocked at 34°C for 60 min; g, comparison of expression levels of the *wp tsl* mutant strain kept at 25°C and heat-shocked at 34°C for 120 min; h, expression levels of both strains kept at 25°C and the *wp tsl* mutant strain heat-shocked at 34°C for 60 min; i, expression levels of both strains kept at 25°C and the *wp tsl* mutant strain heat-shocked at 34°C for 120 min. The “SNP” column includes both synonymous and nonsynonymous substitutions.

**Fig. 2.**
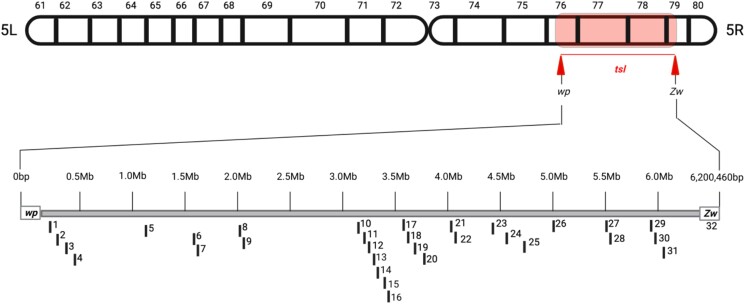
Graphical representation of the *Ceratitis capitata tsl* genomic region including the in silico position of the 32 DE genes. Gene numbering is according to Fig. 1.

The number of RNA-Seq reads obtained for the different embryonic stages, and thermal treatments of the wild-type Benakeion and the mutant *wp tsl* strains are presented in Supplementary Table 2. The results show an average of 51,981,830 reads/sample with a percentage mapping rate of more than 75% on the Ccap 3.2.1 genome. Following 60 and 120 min of heat-shock at 34°C, 32 DE genes were observed comparing the wild-type Benakeion and the *wp tsl* mutant strains (Figs. 1 and 2).

### Genomic analysis—detection of polymorphisms

The *wp tsl* (male and female) and VIENNA 7 (female) Illumina NGS whole genome sequencing read pairs (Supplementary Table 3) were mapped to the *tsl* genomic region extracted from Ccap3.2.1 resulting in a coverage of 98.4% and a pairwise identity of 92.6%. Using the mapped Illumina genome sequence data and the Geneious prime tool “*Find Variations/SNPs*”, 2,782 SNPs, 220 insertions and 65 deletions in coding regions and 40,072 SNPs, 9,644 insertions and 2,352 deletions in noncoding (NC) regions were identified in the *tsl* genomic region. Most of the polymorphisms were SNPs and were detected in the NC sequences (72.7%), which included intronic sequences and sequences up to 1,000 bp upstream of the starting codon of the genes. It is worth noting that most detected indels were single nucleotides and were found in highly repetitive AT-rich regions.

Total polymorphism frequency distribution plots in coding (C) and NC regions showed (Supplementary Fig. 1) a nonuniform pattern along the *tsl* genomic region which was confirmed by three different ratios: (1) the number of polymorphisms found in noncoding regions (PNC) divided by the number of genes; (2) the number of polymorphisms found in coding regions (PC) divided by the number of genes; and (3) the number of PNC divided by the number of PC (Table 1).

**Table 1.** Distribution of polymorphisms in the medfly *tsl* genomic region.

Position in *tsl* genomic region (Mb)	0–0.5	0.5–1.0	1.0–1.5	1.5–2.0	2.0–2.5	2.5–3.0	3.0–3.5	3.5–4.0	4.0–4.5	4.5–5.0	5.0–5.5	5.5–6.0	6.0–6.2	Total
Genes	46	22	43	34	33	29	54	49	58	48	55	70	20	561
Polymorphisms (NC)*^a^*	3,223	6,759	6,251	5,736	6,541	925	3,507	4,725	2,965	1,950	4,657	3,760	1,069	52,068
Polymorphisms (C)*^b^*	273	158	376	170	158	6	260	282	108	74	754	402	46	3,067
NC/genes	70.1	307.2	145.4	168.7	198.2	31.9	64.9	96.4	51.1	40.6	84.7	53.7	53.5	
C/genes	5.9	7.2	8.7	5.0	4.8	0.2	4.8	5.8	1.9	1.5	13.7	5.7	2.3	
NC/C	11.8	42.8	16.6	33.7	41.4	154.2	13.5	16.8	27.5	26.4	6.2	9.4	23.2	

NC, polymorphisms (SNPs and indels) in the noncoding region.

C, polymorphisms (SNPs and indels) in the coding region.

The 3,067 polymorphisms detected in the coding sequences were distributed among 322 genes, representing 57.4% of the 561 genes in the *tsl* genomic region. The polymorphisms observed in the coding sequences were classified into transition and transversion point mutations, as well as synonymous and nonsynonymous mutations. Most of the SNPs, 1,806 or 64.9%, resulted in transitions, while only 976 (35.1%) were transversions. In addition, synonymous mutations were slightly higher than nonsynonymous mutations (1,555 vs 1,512 or 50.7% vs 49.3%), at a ratio of 1.03. Moreover, 1,229 out of the 3,067 polymorphisms detected in coding regions resulted in amino acid changes, while 248 in frameshift mutations.

Using the same procedure, a total of 320 SNPs, 3 insertions, and 1 deletion were detected in the 32 DE genes. Using MEGABLAST, all 324 polymorphisms were confirmed by the 10X genome sequence data of the wild-type EgII and the *wp tsl* mutant strain (Supplementary Table 4).

### *Drosophila melanogaster* heat-sensitive genes: medfly orthologs and detection of polymorphisms

**Fig. 3.**
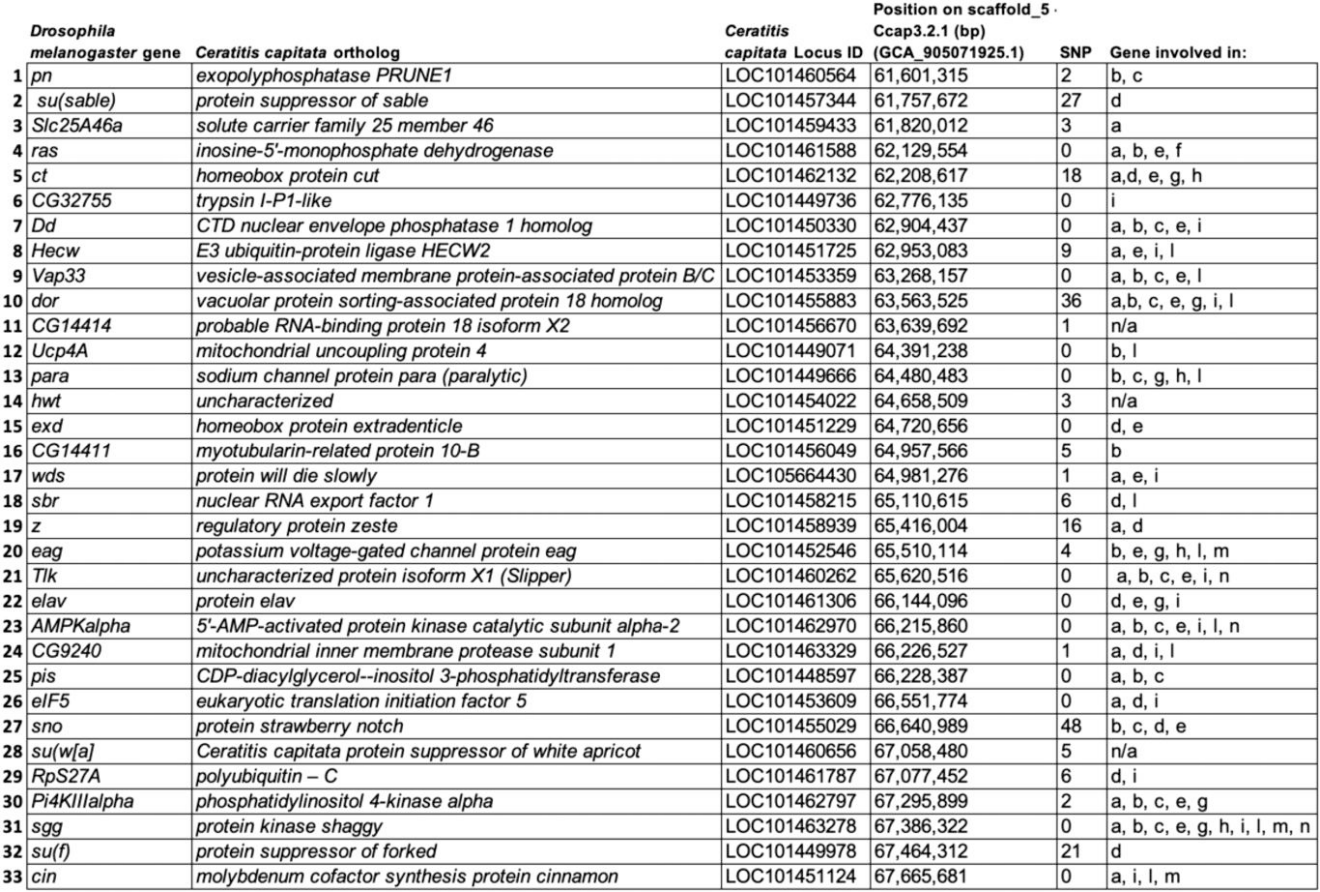
*Drosophila melanogaster heat-sensitive* genes and their orthologs in the *Ceratitis capitata tsl* genomic region. *Drosophila melanogaster* gene name and locus, *C. capitata* ortholog gene name, locus, and position in the reference genome sequence, SNPs detected in the *wp tsl* mutant strain coding sequences, and gene function in *Drosophila* are shown. All SNPs were confirmed by the 10X Genomics medfly genome sequence data. a, cell organization/biogenesis; b, response to stimulus; c, signalin; d, gene expression; e, development; f, small molecule metabolism; g, reproduction; h, nervous system process; i, protein metabolism; l, transport/localization; m, behavior; n, cell cycle/proliferation; n/a, not available. The “SNP” column includes both synonymous and nonsynonymous substitutions.

Screening the FlyBase website with the keyword “heat sensitive genes”, a total of 1,144 *D. melanogaster* heat-sensitive genes were found: 426 of them are located on chromosome 2, 480 on chromosome 3, 15 on chromosome 4, 219 on chromosome X, and 1 on chromosome Y. *Drosophila melanogaster* heat genes protein sequences were searched via tblastn, using Geneious Prime 2022.1.1, against *C. capitata tsl* genomic region resulting in the discovery of 33 orthologous genes: for 32 of them, the *D. melanogaster* gene are located on chromosome X (which is known for its synteny to the right arm of the medfly chromosome 5) and for just one, the gene is present on chromosome 2. Coding region polymorphism calling, using NGS data from the medfly *tsl* mutant strains, identified a total of 222 SNPs (37 of which resulted in amino acid changes and 13 in frameshift mutations), 12 insertions and 3 deletions in 19 genes of the medfly *tsl* genomic region in the mutant strain (Fig. 3). Using the 10X Genomics medfly genome sequence data, all 237 polymorphisms were confirmed.

### In situ hybridization of *tsl* candidate genes

**Fig. 4.**
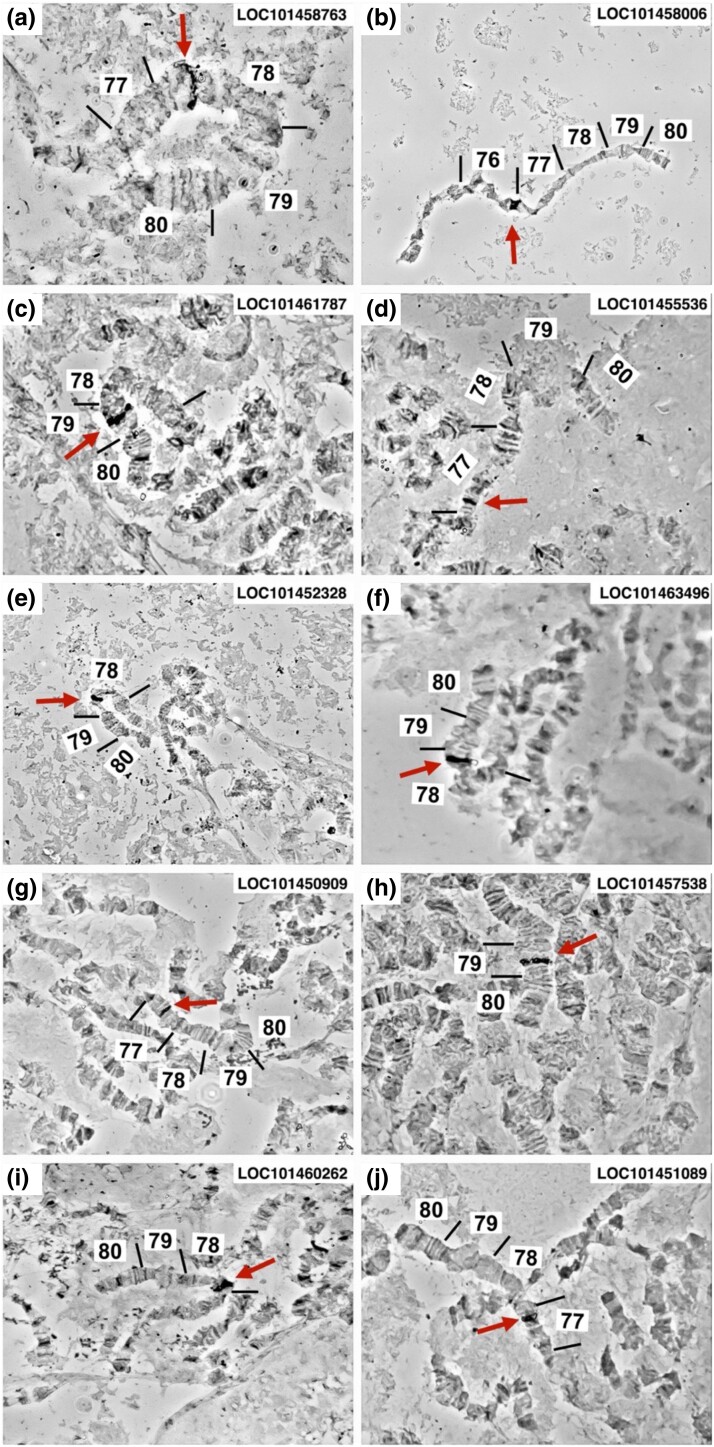
In situ hybridization of *tsl* candidate genes on the right arm of the medfly polytene chromosome 5 (5R). Nuclei with the in situ hybridization signal (arrow) are presented for the following 10 genes: a) *LOC101458763*, b) *LOC101458006*, c) *LOC101461787*, d) *LOC101455536*, e) *LOC101452328*, f) *LOC101463496*, g) *LOC101450909*, h) *LOC101457538*, i) *LOC101460262*, and j) *LOC101451089*, respectively.

**Fig. 5.**
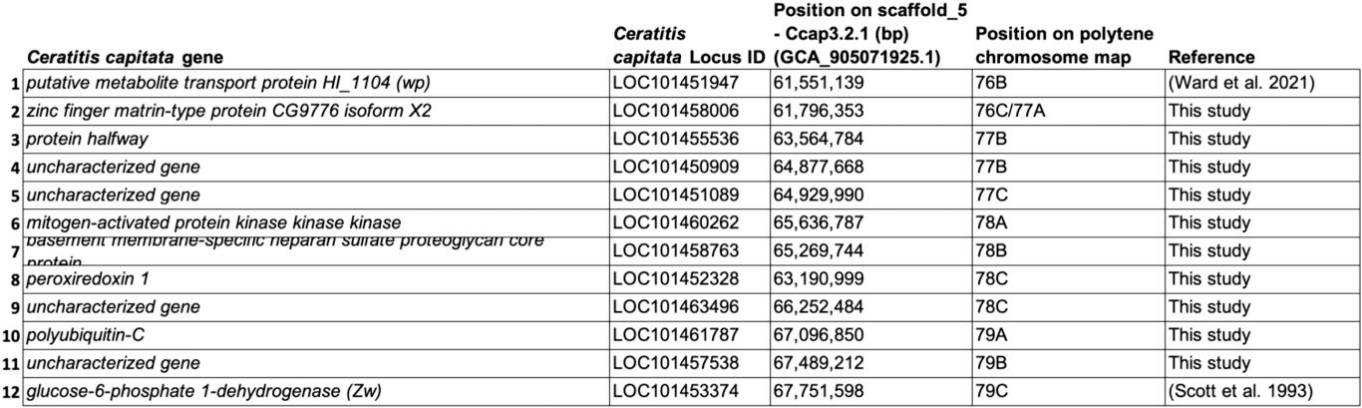
In situ hybridization results of 10 *Ceratitis capitata tsl* candidate genes. In addition to the in situ localization results on the medfly polytene chromosome 5, the position of the genes on medfly chromosome 5 based on published assembly (Ccap3.2.1_scaffold_5) is indicated. The already-known localization of the *wp* and *Zw* genes is also presented.

The results of the in situ hybridization analysis of 10 randomly selected *tsl* candidate genes are presented in Figs. 4 and 5 and Supplementary Fig. 2. All hybridizations provided a clear and unique signal confirming that these are single-copy genes (Fig. 4). In addition, the results of this analysis confirmed that the order of the genes tested largely agrees with the recently published assembly of the *tsl* genomic region, spanning the area from the *wp* gene to the *Zw* gene (6,200,460 bp long), except for one gene (*LOC101452328*; *peroxiredoxin 1*) (Figs. 4 and 5 and Supplementary Fig. 2) (Papanicolaou *et al*. 2016; Ward *et al*. 2021).

## Discussion

The *tsl* gene has been used together with the *wp* gene as a selectable marker for the construction of the most successful GSS developed so far, the medfly VIENNA 8 GSS (Rössler 1979; Franz *et al*. 2021). This GSS is currently used in SIT applications against this major agricultural pest in almost all continents (Knipling 1955; Cayol *et al*. 2002; Millo *et al*. 2003; Porras-Reyes 2007; Zavala Lopez and Enkerlin 2017; Enkerlin 2021; Klassen and Vreysen 2021). It has been suggested that discovering the genes responsible for these two phenotypes could open the way for their generic use in constructing GSS against other insect pests and disease vectors (Caceres 2002; Franz *et al*. 2021). As the *wp* gene was recently discovered (Ward *et al*. 2021), the research efforts have focused on discovering the medfly *tsl* gene.

In the present study, we characterized the medfly *tsl* region using genomic, transcriptomic, bioinformatic, and cytogenetic analyses to identify candidate *tsl* genes for downstream functional analysis and potential linkage with the temperature-sensitive lethal phenotype reported in this species (Franz *et al*. 2021). Analysis of the available genetic and genomic information suggests that the *tsl* gene is in the *wp–Zw* (*tsl*) region which contains 561 genes. Earlier studies used deletion mapping and cytogenetic analysis to locate the *tsl* gene downstream of the *wp* locus, in the 59B–61C region of the trichogen polytene chromosome map. The most likely location of the gene is in the 60C–61B region, which corresponds to the 77–78 region of the polytene chromosome map, and is very close to the *Sergeant-2* gene (Kerremans and Franz 1994; Niyazi *et al*. 2005). However, since the insect lines used in the deletion mapping are no longer available, the comparative analysis of wild-type and *tsl* mutant strains was pursued.

Comparative transcriptomic analysis showed that 32 genes are DE between the wild-type and the *wp tsl* mutant strain upon a specific thermal treatment of embryos. The list of DE genes includes genes which are involved in diverse functions such as cell organization/biogenesis, development, response to stimulus, signaling as well as gene expression (Supplementary Table 5). Comparative genomic analysis of wild-type and mutant strains revealed the presence of 324 polymorphisms (320 SNPs, 3 insertions and 1 deletion) in the coding region of 24 DE genes. Interestingly, the orthologs of two of the 32 DE genes, *LOC101460262* and *LOC101448597*, have been associated with heat-sensitive phenotypes in *D. melanogaster* (Polaski *et al*. 2006; Wang and Montell 2006). The ortholog of *LOC101460262* is known as *slipper* in *D. melanogaster.* It has mainly been involved in functions such as system development, signal transduction, response to heat, transmembrane receptor protein tyrosine kinase signaling pathway, and regulation of the embryonic development (Harden 2002; Martin and Wood 2002; Sathyanarayana *et al*. 2002; Stronach and Perrimon 2002; Sathyanarayana *et al*. 2003; Ishimaru *et al*. 2004; Polaski *et al*. 2006; Baril *et al*. 2009; Gonda *et al*. 2012). The ortholog of *LOC101448597* is known as *phosphatidylinositol synthase* (*pis*) in *Drosophila*. It has been shown to participate in basement membrane organization, phototransduction, and phosphatidylinositol biosynthetic processes (Wang and Montell 2006; Gaudet *et al*. 2011; Devergne *et al*. 2014), as well as being responsible for lethality during the embryonic stage when mutated (Wang and Montell 2006; Liu *et al*. 2014).

Comparative genomic analysis between the wild-type and the *wp tsl* mutant strain identified many polymorphisms (SNPs and indels) in the coding region of 322 out of the 561 genes in the *tsl* region. Such polymorphisms, with or without transcriptomic data, can be exploited in genome-wide associated studies to assess genotype–phenotype associations (Rodrigues *et al*. 2016; Sim and Geib 2017; Sim *et al*. 2017; Mhoswa *et al*. 2020; Ward *et al*. 2021). It is also important to note that a significant number of polymorphisms was detected in NC regions because, as it has been shown in several studies, not only single point mutations (Aumann *et al*. 2020; Choo *et al*. 2020) or combination of different single point mutations (Lovato *et al*. 2009) in coding regions but also deletions (Kellis Jr *et al*. 1989; Sandberg and Terwilliger 1989; Lim *et al*. 1992; Varadarajan and Richards 1992; Matthews 1995; Shi *et al*. 2019) and insertions (Kellis Jr *et al*. 1989; Sandberg and Terwilliger 1989; Lim *et al*. 1992; Varadarajan and Richards 1992; Matthews 1995; Mela *et al*. 2009; Srinivas and Cronan 2017) located in intergenic and promoter regions can be responsible for temperature-sensitive lethal phenotypes. In addition, it was recently shown in the yeast *Saccharomyces cerevisiae* (Shen *et al*. 2022) that synonymous mutations, which do not alter protein sequences, may affect gene expression levels in a way similar to nonsynonymous mutations. This implies that each of the mutations identified in the *wp tsl* mutant strain could potentially be associated with the tsl phenotype.

Using a text mining approach, 1,144 known *D. melanogaster* heat-sensitive genes were retrieved from FlyBase, with 33 having orthologs in the medfly *tsl* genomic region. Polymorphisms, including SNPs and indels were found in 19 of the 33 medfly orthologs, expanding the list of candidate genes for a tsl phenotype. Among these, three *D. melanogaster* genes exhibit very interesting phenotypes: the gene *protein will die slowly* (*wds*; medfly ortholog *LOC105664430*), the gene *nuclear RNA export factor 1* (*sbr*; medfly ortholog *LOC101458215*) and the gene *deep orange* (*dor*; medfly ortholog *LOC101455883*).

They are involved in various cellular processes, including cell organization/biogenesis, development and protein metabolism, transport/localization, gene expression, cellular component organization, and response to a stimulus. Homozygous mutations in these genes lead to embryonic, larval, or pupal lethality at moderate or high temperatures (25°C for *wds* and 29°C for *sbr* and *dor*) (Lindsley and Grell 1968; Shannon *et al*. 1972; Belyaeva *et al*. 1982; Zhimulev *et al.* 1982; Sevrioukov *et al*. 1999; Wilkie *et al*. 2001; Sriram *et al*. 2003; Medioni and Noselli 2005; Suganuma *et al*. 2008; Wilkin *et al*. 2008; Gaudet *et al*. 2011; Pascual-Garcia *et al*. 2014; Takats *et al*. 2014; Guo and Jin 2015; Lorincz *et al*. 2016; Zhu *et al*. 2017).

Several *Dm* genes such as *shibire, notch, pale, transformer-2, downstream of Raf1* and *RNA polymerase II 215kD* subunit were considered potential *tsl* candidate genes as they exhibit temperature-sensitive lethal phenotype due to single point mutations (Nguyen *et al*. 2021). Still, none of them was found in the medfly *tsl* genomic region. It should be noted, however, that one of them, *shibire (shi)*, was localized downstream of the *Zw* gene by in situ hybridization (data not shown). Sequence analysis of *Ccshi* in wild-type and *tsl* mutant strains showed no evidence of polymorphisms, thus allowing us to remove this gene from the list of *tsl* candidate genes (data not shown). However, the *shi* gene can be potentially exploited to induce a tsl-like temperature-sensitive lethal phenotype (Choo *et al*. 2020; Nguyen *et al*. 2021).

As the entire in silico analysis of the present study was performed using the *C. capitata* 3.2.1 genome (Ccap3.2.1; accession GCA_905071925.1), it was important to confirm that the published assembly in the *tsl* genomic region is as accurate as possible. Using in situ hybridization analysis and a set of 10 genes spanning the *tsl* region, the localization of the genes tested was largely in agreement with the assembly of the *tsl* genomic region (Papanicolaou *et al*. 2016; Ward *et al*. 2021) except for one gene (*LOC101452328*, *peroxiredoxin 1*). According to earlier studies, the *tsl* gene is localized in the region 77–78 of the polytene chromosome map (Kerremans and Franz 1994; Niyazi *et al*. 2005). Our data show that the *peroxiredoxin 1* is mapped in 78C, which is close to the predicted localization of the *tsl* gene. This further emphasizes the importance of having a high-quality reference genome available.

Taken together, the present study unraveled the genomic and transcriptomic differences in the *tsl* genomic region of wild-type and *tsl* mutant strains. While it may not have produced a significant reduction in the target region, the study did identify several promising candidate genes that may be involved in the induction of temperature-sensitive lethal phenotypes. The genetic basis of the tsl phenotype remains unknown and can be due to one or more, protein or not, coding genes; the causal mutation(s) can be either in the coding or the regulatory region; the mutation(s) can be a single (or multiple) nucleotide (synonymous or nonsynonymous) substitution, indel(s) or chromosomal rearrangements (for example, a small size inversion). Clearly, the discovery of the *tsl* gene and its causal mutation(s) poses a significant challenge. However, this study's integrated approach has generated a list of potential candidate genes that could be linked to this phenotype. Downstream functional analysis based on genome editing tools is required to identify if any of these candidates is the*tsl* gene. If the causal mutation is an SNP, the functional verification will be based on inducing the same mutation in a wild-type strain and/or reconstructing the wild-type allele of the gene in a mutant strain followed by the necessary temperature sensitive lethal tests on the established genome edited lines to assess their temperature sensitivity. This process may also lead to the discovery of novel genes involved in the expression of temperature-sensitive lethal phenotypes.

## Supplementary Material

jkad074_Supplementary_Data

## Data Availability

Sequencing files can be found at NCBI under the following project numbers: PRJEB57574. Supplemental material available at G3 online.
