# Supplementary material for: Genomic and cytogenetic analysis of the Ceratitis capitata temperature-sensitive lethal region
Source: G3 (Bethesda). 2023 Mar 29;13(6):jkad074. doi: 10.1093/g3journal/jkad074 (PMC10234411; doi:10.1093/g3journal/jkad074)
Supplement: jkad074_Supplementary_Data [file jkad074_supplementary_data.docx]

Supplementary Material

**Genomic and cytogenetic analysis of the *Ceratitis capitata* *temperature-sensitive lethal* region**

**Germano Sollazzo^1,2^, Georgia Gouvi^1,3^, Katerina Nikolouli^1^, Roswitha A. Aumann^2^, Haig Djambazian^4^, Mark A. Whitehead^5^, Pierre Berube^4^, Shu-Huang Chen^4^, George Tsiamis^3^,** **Alistair C. Darby^5^, Jiannis Ragoussis^4^, Marc F. Schetelig^2,*^ and Kostas Bourtzis^1,*^**

^1^ Insect Pest Control Laboratory, Joint FAO/IAEA Centre of Nuclear Techniques in Food and Agriculture, Friedensstrasse 1, 2444 Seibersdorf, Austria.

^2^ Justus-Liebig-University Gießen, Institute for Insect Biotechnology, Department of Insect Biotechnology in Plant Protection, Winchesterstr. 2, 35394 Gießen, Germany.

^3^ Laboratory of Systems Microbiology and Applied Genomics, Department of Sustainable Agriculture, University of Patras, 2 G. Seferi St., 30100, Agrinio, Greece.

^4^ McGill University Genome Centre, McGill University, Montreal, QC, Canada.

^5^ Centre for Genomic Research, Institute of Integrative Biology, The Biosciences Building, Crown Street, L69 7ZB Liverpool, United Kingdom.

*Corresponding author:

Email: K.Bourtzis@iaea.org; Marc.Schetelig@agrar.uni-giessen.de

Keywords:

Mediterranean fruit fly, sterile insect technique, genetic sexing strain, white pupae, Tephritidae

**Table S1**. Primers used for the preparation of probes for the *in situ* hybridization analysis.

| ***Ceratitis capitata* gene** | **Forward (F) and reverse (R) primers used for probes in *in situ* hybridization** |
| --- | --- |
| *zinc finger matrin-type protein CG9776 isoform X2* | F: ATTGGATCGGGCATAAGCTCT |
|  | R: ACCTCCAGTGATTCCTCCTCA |
| *protein halfway* | F: TGCTCGTCAAATTATTCTTCGAGAT |
|  | R: TCACTCCTTCTCTTCGGACTAAC |
| *uncharacterized LOC101450909* | F: CGATCCCGATGCCACACAG |
|  | R: CGTCGCATACGTCACTCACT |
| *uncharacterized LOC101451089* | F: AGACATAAACCGACGGCAAC |
|  | R: TGCTAAAGTGGACAGCGTCA |
| *mitogen-activated protein kinase kinase kinase* | F: GTTTGTCGGTTTTCAGGCGT |
|  | R: GTGGCGCAAGTTCAGAAGAG |
| *basement membrane-specific heparan sulfate proteoglycan core protein* | F: AATCGCCAGCGGTATTGTGA |
|  | R: GCACTGCCCGGTGGTATTAT |
| *peroxiredoxin 1* | F: TTGCAGCACATCCCACATTG |
|  | R: TGGCGTGAAATGACGGTACA |
| *uncharacterized LOC101463496* | F: CACTCAGACACAAGCGACCT |
|  | R: CGCGCCGCAGCTATAATAAC |
| *polyubiquitin-C* | F: GCACTGCCCGGTGGTATTAT |
|  | R: ACGTTGTTGATCTGGGGGAA |
| *uncharacterized LOC101457538* | F: TTGCAGCACATCCCACATTG |
|  | R: TGGCGTGAAATGACGGTACA |

**Table S2.** RNA-seq results of the *Ceratitis capitata* wild-type Benakeion and *wp tsl* mutant strains (BioProject No PRJEB57574).

| **Strain** | **Name** | **Number of reads (bp)** |
| --- | --- | --- |
| Benakeion | Ben_25_R1 | 58,124,080 |
| Benakeion | Ben_25_R2 | 37,604,618 |
| Benakeion | Ben_25_R3 | 35,035,902 |
| Benakeion | Ben_60_R1 | 99,190,658 |
| Benakeion | Ben_60_R2 | 43,753,856 |
| Benakeion | Ben_60_R3 | 34,902,306 |
| Benakeion | Ben_120_R1 | 93,219,632 |
| Benakeion | Ben_120_R2 | 40,344,904 |
| Benakeion | Ben_120_R3 | 28,550,468 |
| *wp tsl* | tsl_25_R1 | 79,382,592 |
| *wp tsl* | tsl_25_R2 | 44,741,000 |
| *wp tsl* | tsl_25_R3 | 37,458,356 |
| *wp tsl* | tsl_60_R1 | 87,185,310 |
| *wp tsl* | tsl_60_R2 | 47,286,386 |
| *wp tsl* | tsl_60_R3 | 46,189,739 |
| *wp tsl* | tsl_120_R1 | 48,851,950 |
| *wp tsl* | tsl_120_R2 | 25,631,810 |
| *wp tsl* | tsl_120_R3 | 48,219,365 |

Ben_25 and tsl_25 = Benakeion wild-type strain and *wp tsl* mutant strain kept at 25 °C.

Ben_60 and tsl_60 = Benakeion wild-type strain and *wp tsl* mutant strain kept at 34 °C for 60 minutes.

Ben_120 and tsl_120 = Benakeion wild-type strain and *wp tsl* mutant strain kept at 34 °C for 120 minutes.

R1, R2, R3 = three replicates were analyzed per strain and treatment.

**Table S3.** Illumina NGS results of the *Ceratitis capitata* VIENNA 7 GSS and *wp tsl* mutant strain (BioProject No PRJEB57574).

| **Strain** | **Total reads** | **Total reads bases (bp)** |
| --- | --- | --- |
| *wp tsl* (male) | 58,541,784 | 8,839,809,384 |
| *wp tsl* (female) | 59,122,524 | 8,927,501,124 |
| VIENNA 7 (female) | 45,703,818 | 6,901,276,518 |

**Figure S1.** Non-coding (A) and coding (B) region polymorphisms frequency distribution plot of the medfly *wp-Zw* genomic region. The horizontal axis shows the length of *wp-Zw* region (bp) while the vertical axis indicates the number of polymorphisms.

**Table S4.** 10X Genomics sequencing results of the *Ceratitis capitata* wild-type EgII and *wp tsl* mutant strains (BioProject No PRJEB57574).

| **Strain** | **Total reads** | **Total reads bases (bp)** |
| --- | --- | --- |
| EgII (male) | 188,183,315 | 56,831,361,130 |
| EgII (female) | 271,196,767 | 81,901,423,634 |
| *wp tsl* (male) | 207,972,616 | 62,807,730,032 |
| *wp tsl* (female) | 175,767,398 | 53,081,754,196 |

**
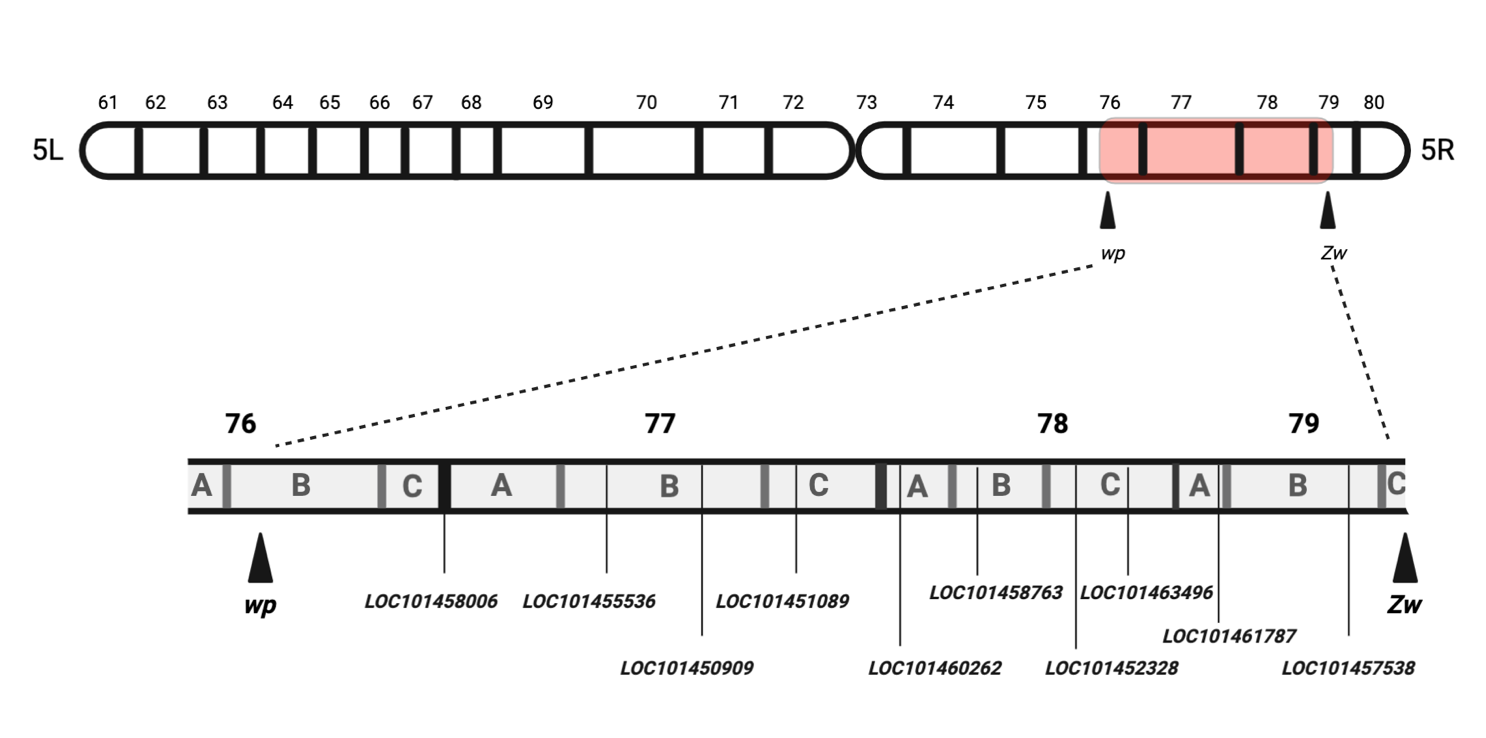
**

**Figure S2.** Schematic representation of *Ceratitis capitata* chromosome 5 showing the *tsl* genomic region [25, 27] and the localization of selected *tsl* genes by *in situ* hybridization on salivary gland polytene chromosomes.

**Table S5.** ***Ceratitis capitata* differentially expressed genes located in the *tsl* region.** The function of the *Drosophila melanogaster* orthologs is also shown.

| **Gene** | **Gene involved in:** |
| --- | --- |
| *zinc finger matrin-type protein CG9776* | Gene expression |
| *uncharacterized protein* | n/a |
| *thyroid adenoma-associated protein homolog* | Transport/localization, gene expression |
| *microtubule-associated protein futsch* | Cell organization/biogenesis, transport/localization, development, nervus system process, behavior, response to stimulus |
| *flocculation protein FLO11* | n/a |
| *small G protein signaling modulator 2* | Transport/localization |
| *peroxiredoxin 1* | Development, reproduction, immune system, response to stimulus |
| *protein penguin* | Development, gene expression, protein metabolism |
| *b(0,+)-type amino acid transporter 1* | Transport/localization |
| *coiled-coil domain-containing protein* | n/a |
| *uncharacterized protein* | n**/**a |
| *mucin-5AC isoform X1, mucin-5AC* | n/a |
| *uncharacterized protein* | Cell organization/biogenesis, transport/localization, development, response to stimulus, signaling |
| *uncharacterized protein* | n/a |
| *probable methylmalonate-semialdehyde dehydrogenase* | Small molecule metabolism |
| *uncharacterized protein* | n/a |
| *not found* | n/a |
| *retinol dehydrogenase 12* | n/a |
| *uncharacterized protein* | Cell organization/biogenesis, transport/localization, development, nervus system process, behavior, response to stimulus, signaling |
| *basement membrane-specific heparan sulfate proteoglycan core protein* | Cell cycle proliferation, cell organization/biogenesis, development, response to stimulus, signaling |
| *uncharacterized protein* | Cell organization/biogenesis, development, reproduction, response to stimulus, signaling, protein metabolism |
| *transcription factor Adf-1* | Gene expression |
| *amyloid-beta-like protein* | Cell organization/biogenesis, development, nervus system process, behavior, response to stimulus |
| *arginase-1* | Small molecule metabolism |
| *CDP-diacylglycerol--inositol 3-phosphatidyltransferase* | Cell organization/biogenesis, response to stimulus, signaling |
| *uncharacterized protein* | n/a |
| *inositol oxygenase* | Small molecule metabolism |
| *uncharacterized protein* | n/a |
| *uncharacterized protein* | Cell organization/biogenesis, gene expression, protein metabolism |
| *alkaline phosphatase* | Response to stimulus |
| *uncharacterized protein* | n/a |
| *glucose-6-phosphate 1-dehydrogenase* | Small molecule metabolism |

n/a = not available
